# Capgras Syndrome and Unilateral Spatial Neglect in Nonconvulsive Status Epilepticus

**DOI:** 10.3233/BEN-2008-0210

**Published:** 2009-06-01

**Authors:** L. Christine Turtzo, Jonathan T. Kleinman, Rafael H. Llinas

**Affiliations:** ^1^Department of NeurologyUniversity of ConneticuttStorrsCTUSA; ^2^Department of NeurologyJohns Hopkins Medical CenterBaltimoreMDUSA; ^3^School of MedicineStandford UniversityPalo AltoCAUSA

## Abstract

Nonconvulsive status epilepticus can manifest as personality changes and psychosis. We report an 87-year-old right-handed male presenting with both Capgras syndrome and severe unilateral spatial neglect during nonconvulsive status epilepticus. After treatment of his seizures, his Capgras syndrome and hemispatial neglect resolved. This case illustrates a report of the confluence of Capgras syndrome and documented hemispatial neglect in nonconvulsive status epilepticus only reported once previously [1].

## 1. Introduction

Nonconvulsive status epilepticus (NCSE) is defined as ongoing or intermittent electroencephalographical seizure activity for a minimum of 30 minutes, associated with cognitive or behavioral changes, in the absence of convulsive clinical manifestations [2]. The non-specific manifestations of NCSE, which may include confusion, behavioral changes, personality change, and psychosis, as well as the focal neurological presentation such as aphasia [3,4] make EEG findings crucial for its diagnosis when suspected [5–7]. While NCSE is more common in the elderly than in the young [8], its diagnosis in the elderly is often delayed while other etiologies for delirium are pursued [9,10]. Considerable debate exists regarding the diagnosis and prognosis of NCSE, although elderly patients, especially those with comorbid medical conditions, appear to have higher mortality associated with NCSE [2]. In this case report, we describe an 87-year-old patient who manifested concurrent hemispatial neglect and Capgras syndrome in conjunction with NCSE.

## 2. Case report

An 87-year-old right-handed male with a history of diabetes, hypertension, hypercholesterolemia, mild cognitive impairment, and bradycardia presented with an episode of slurred speech and blindness. At baseline he was able to perform his own activities of daily living. He was in his usual state of health until the evening prior to presentation, when he experienced what he described as a "blinding flash of light", was unable to respond to his wife's request to turn off the oven and had to 'feel [his] way' to the bedroom. The next morning he had a headache, slurred speech, and difficulty walking, and his family brought him to the emergency department.

On initial examination he was afebrile and normotensive, and in no acute distress. He was oriented to person and place only, with intact comprehension and language. He had good concentration and no confusion was present. He had left-sided neglect, an apparent left visual field cut, and left arm weakness 4/5. He was admitted with a presumptive diagnosis of right-sided infarct, but his brain MRI showed no acute lesions consistent with stroke, and no evidence of old injury (Fig. 1).

Additional testing of hemispatial neglect was performed, including the following: copy a scene of two

ISSN 0953-4180/08/$17.00 © 2008 – IOS Press and the authors. All rights reserved

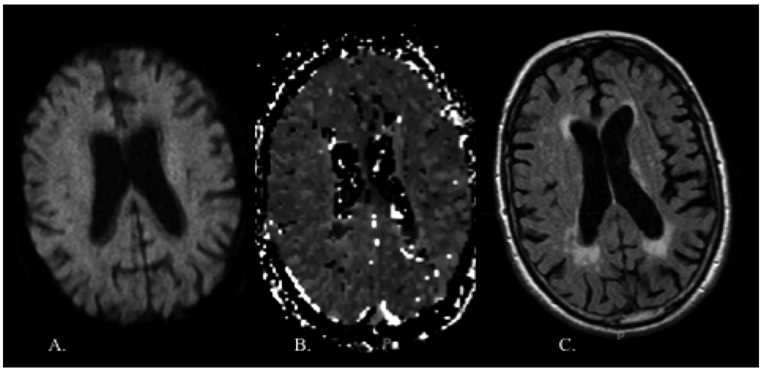

Fig. 1. A) Diffusion-weighted (DWI) MRI while patient was symptomatic shows no acute stroke in right hemisphere B) Perfusion time to peak (TTP) MRI scan shows symmetric cerebral perfusion in the right hemisphere. C) FLAIR T2 weighted MRI shows no edema, focal atrophy or mass effect in right hemisphere.

trees, a house, and a fence [11]; modification of the line cancellation test [12]; line bisection 270 mm line, in which the page was presented 45° to the left and 45° to the right of the midsagittal plane and at the midsagittal plane of the viewer (25–30 cm from the trunk); clock copy; reading words; reading sentences; and reading words written vertically. For each test, we report the number of errors (or percentage deviation from the midpoint on the line bisection test).

The patient made two types of errors on oral reading; neglecting the entire column of words on the left (15), and the left side of the words he attended to (14/15). The patient attended to only one sentence (1/5) and read, "upstairs," when the sentence stated, *Before you go, please take this upstairs.* In contrast when there was no left to right component of reading (vertical reading), the patient made *no* errors. While copying the scene, the patient missed all but the shapes furthest to the right side of the page. While drawing the clock the patient drew a circle, then next to it wrote "3:45." The deviation from midline on line bisection increased as the page was moved from right to left hemispace (86.4% error) (Fig. 2A). The patient displayed significant left neglect on line cancellation, and associated perseveration on the lines he did cancel (Fig. 2B). The patient had severe tactile extinction, missing all twelve double simultaneous stimulations. The patient did not attend to single stimuli in his left visual field mimicking a left visual field cut.

Two days after admission the patient became more confused and agitated, and lost the ability to recognize his family members. On catching sight of his daughter, he recognized that she resembled his daughter, but insisted that she was someone impersonating his daughter. He stated emphatically, "Now I have proof of the existence of witches." He also failed to recognize his wife, insisting that she too had been replaced by someone else, stating "You changed her, why did you take her from me?"

An EEG was performed, which was markedly abnormal. During the 26 minute recording, a buildup of high frequency 15 Hz 5 to 10 MCV sharp activity arose over the right parieto-temporal occipital region, followed 3 seconds later by right tempero-occipital rhythmic activity at 4 Hz lasting for 200 seconds before subsiding. There was residual 4 to 5 Hz higher voltage theta activity over the right frontal region afterwards, with no return to baseline. Towards the end of the recording the patient experienced another episode of rhythmic electrographic activity similar to the first. Throughout the recording the patient demonstrated no motor or vocal correlates of his electrographic activity, aside from the persistence of his difficulties with vision and neglect.

With his EEG showing 2 electrographic seizures arising from the right temporo-parieto-occipital region without return to baseline between events, the patient was diagnosed with non-convulsive status epilepticus. The patient was loaded with phenytoin, with subsequent maintenance dosing. Once begun on antiepileptic therapy, the patient was able to recognize his family members again, and his left-sided neglect and weakness resolved, with normalization of his EEG findings. After his seizure diagnosis several family members reported that prior to this admission the patient had had several episodes of altered behavior for several hours to days at a time, during which he had been 'out of it' but able to perform his daily activities with minimal assistance. He developed a rash after initiation of phenytoin, and was transitioned to levetiracetam instead.

After discharge, the patient was seen in neurology clinic for followup of his seizures. He developed para-

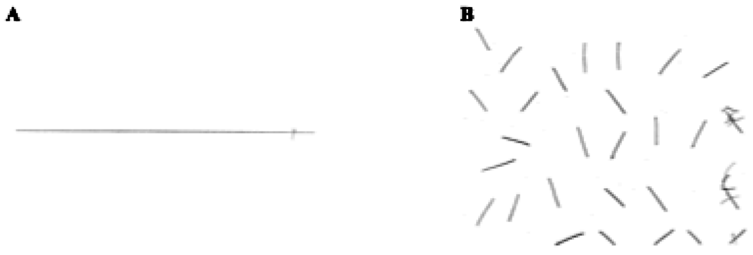

Fig. 2. Patient's performance on tests of hemispatial neglect. A) Line bisection test. B) Line cancellation test.

noid ideation while on levetiracetam, and was transitioned to lamotrigine monotherapy. He had no further seizures as of the time of his last neurology followup. Before follow-up neuropsychiatric testing could be performed, three months status-post discharge he died of MRSA sepsis secondary to septic arthritis of his knee.

## 3. Discussion

The present case illustrates the manifestation of both profound hemispatial neglect and Capgras syndrome in a patient with nonconvulsive status epilepticus. The patient's presentation initially appeared most consistent with a right-hemispheric stroke, given the predominance of hemispatial neglect, although there was no evidence of infarct or other intracranial lesion by neuroimaging. His development of hallucinations and Capgras syndrome led to further evaluation of his confusion by EEG, which documented NCSE. After antiepileptic treatment, his Capgras syndrome, hallucinations, and hemineglect resolved. His neurological exam returned to normal without any evidence of his prior neglect. The line cancellation test was not repeated after resolution of neurological findings.

Unilateral spatial neglect following brain damage is often defined as the inability to attend or respond to space contralateral to the damage, not attributable to a primary sensory or motor deficit [13]. Deficits in spatial representations have been found to be associated with distinct anatomical regions in a study of patients with acute right hemisphere stroke [14]. Spatial neglect has been reported in postictal, but not interictal states [15,16] and induced through electrocortical stimulation mapping [17]. Therefore, cortical dysfunction of any etiology may cause unilateral spatial neglect.

Capgras syndrome is the delusion that familiar persons have been replaced by impostors, and was first described by Pick in 1903 [18] and then later in 1923 by Capgras and Reboul-Lachaux [19]. The prevalence of Capgras syndrome has been reported to be 2.5% in one study of acute psychiatric inpatients, with approximately half of those patients having evidence of some underlying organic disease [20]. Postictal Capgras syndrome has been reported previously in a patient with complex partial seizures of right temporal origin [21] and in a patient with a frontal meningioma [22]. Ictal or perictal Capgras syndrome has been reported in association with disulfiram use [23] and in a patient with tuberous sclerosis and complex partial seizures of frontal lobe origin [24]. Cases of Capgras syndrome have typically been reported in disorders of the nondominant hemisphere or in cases of bifrontal dysfunction [25,26]. The right temporal region specifically the fusiform gyrus has been shown to be activated in PET studies of facial recognition tasks [27]. The parahippocampal region within the temporal lobe is also implicated in this task, is thought of as a conduit from the hippocampus and fusiform gyrus relaying contextual information and visual memory. The raises the questions of dissociation between the ventral and dorsal visual processing streams. Dorsal visual processing stream is located from the occipital lobe to parietal lobe and primarily is responsible for reaching and object location in space and context, damage can result in impaired non-conscious affective recognition. Ventral visual processing stream is located within: posterior inferior-temporal lobe, central inferior temporal lobe, anterior inferior-temporal and subserves visual identification and form representation thus ventral stream damage results in inability to recognize objects or faces. It may be that Capgras syndrome results from damage with the dorsal stream but not damage to the ventral stream. This resulting in patients with Capgras syndrome recognizing faces normally, but they may lack the expected subjective emotional response that face should produce secondary to associated damage to the dorsal stream. Thus they recognize the face but without an appropriate affective response there is a delusion about that person's identity due to the lack of emotional recognition [24,28]. The presence of a hemi neglect plus Capers syndrome is interesting as it confirms the theory that the dysfunction is in the dorsal visual stream with maintained ventral stream function.

The right-handed patient described in this case report had a right temporoparietal epileptogenic focus correlating with the coexistence of his profound left hemispatial neglect and Capgras syndrome. Suppression of his NCSE activity with anticonvulsants led to resolution of these symptoms, suggesting that epileptogenic activity in this region of his nondominant hemisphere was the origin of his behavioral changes.

## Acknowledgements

This work was supported by NINDS RO1NS47691 (Argye E. Hillis).
